# Supplementary material for: Systematic identification of structure-specific protein–protein interactions
Source: Mol Syst Biol. Author manuscript; Available in PMC 2024 Jun 21. (PMC11148107; doi:10.1038/s44320-024-00037-6)
Supplement: Appendix [file EMS196675-supplement-Appendix.pdf]

Table of Contents

Appendix Figure S1. 2
Appendix Figure S2: 3
Appendix Figure S3: 4
Appendix Figure S4: 5
Appendix Figure S5:. 6
Appendix Figure S6:.......... 7
Appendix Table S1: Definition of variable windows for DIA–MS measurements.......... 8

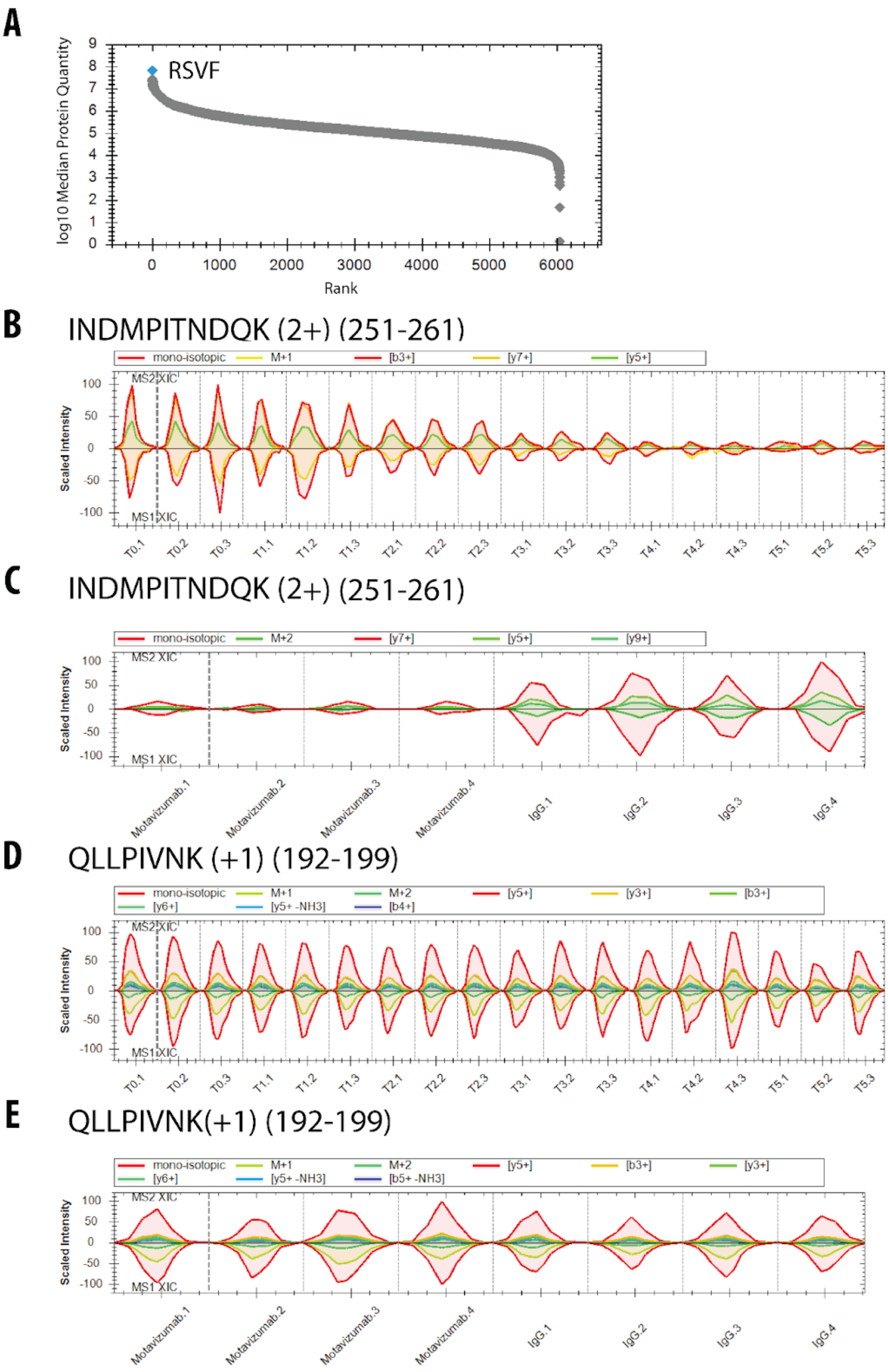

Appendix Figure S1: Further characterization of antibody interactions with RSVF. (A) The plot shows the ranked abundance of all identified proteins (gray) in the experiment analyzing interaction of spiked-in motavizumab and RSVF in cellular lysate. The spiked-in RSVF protein is shown in blue. (B-E) Extracted ion chromatogram (XIC) plots of the indicated peptides: INDMPITNDQK 2+; 251-261 (B, C) and QLLPIVNK +1; 192-199 (D, E). Plots depict the XIC for MS2 (upper row) and the MS1 Isotope Envelope (lower row) across all five conditions (B, D) (T0: untreated sample; T1-T5: treated samples with increasing concentrations of motavizumab) in cellular lysate (n=3 replicates each), and (C, E) in Motavizumab-treated versus IgG control-treated purified RSVF samples (n=4 replicates each).

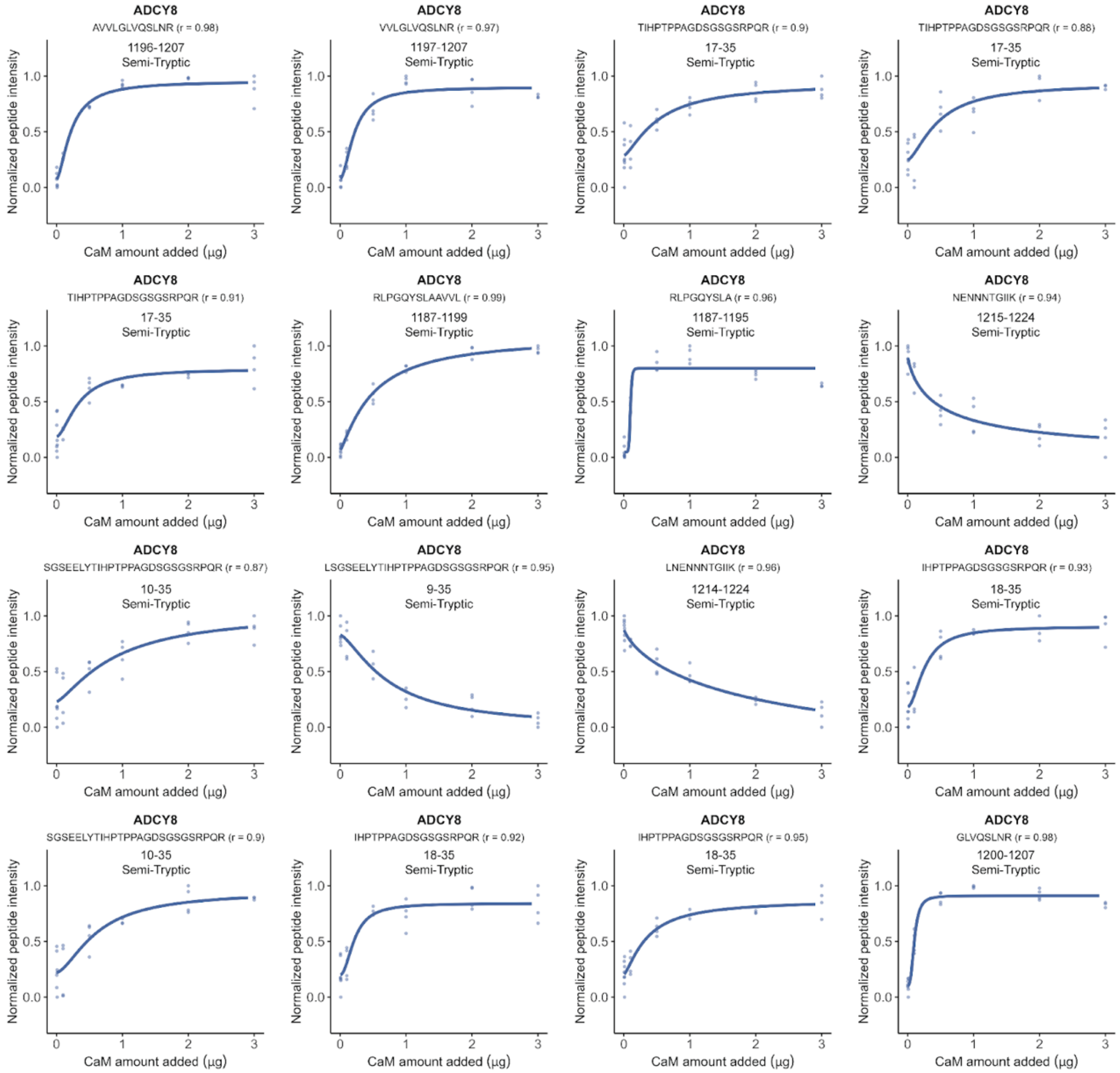

Appendix Figure S2: Dose-response curve of AC8 peptides. Dose-response curves of sixteen LiP peptides with indicated amino acid positions and specificity originating from AC8 show relative peptide intensities proportional to the amount of CaM spiked into crude membranes (n=4 replicates each). Pearson's coefficient (*r*) to a sigmoidal trend of the peptide-intensity response profile is indicated.

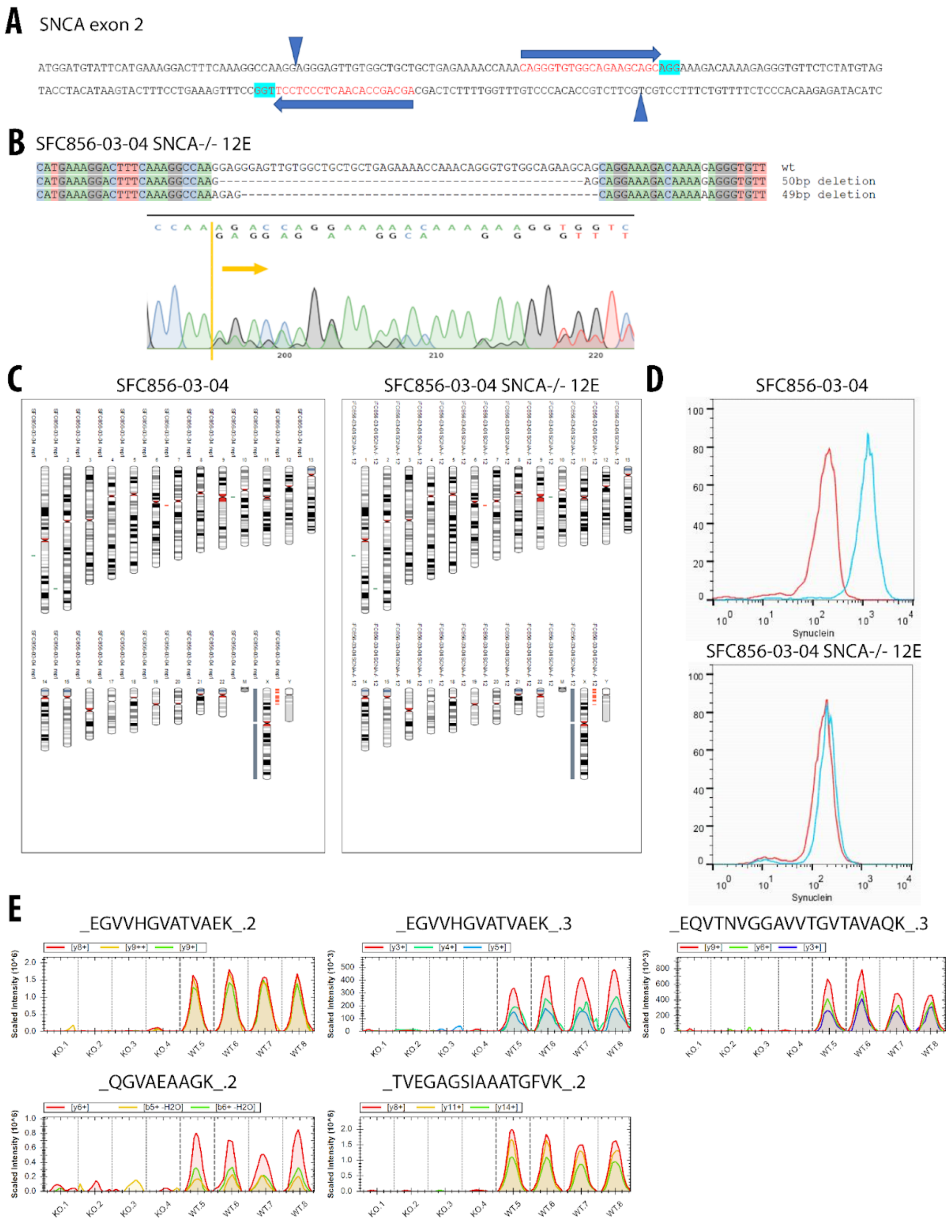

Appendix Figure S3: CRISPR/Cas9-mediated KO of SNCA from human iPSC and characterization of previously unpublished derived KO clone SFC856-03-04 SNCA-/- 12E. (A) The editing strategy for creating SNCA-KO from the healthy donor iPSC line SFC856-03-04, shows the exon 2 sequence section with guide RNA positions (blue arrows), PAM (highlight in turquoise), and expected cut positions (blue triangles). (B) The deconvoluted sequence of both alleles of clone 12E shows out-of-frame repair that will lead to premature translational termination, with the original sequence trace in the lower panel. (C) Karyograms produced from the SNP array show no gross abnormalities in clone SFC856-03-04 SNCA-/- 12E versus the parental iPSC line SFC856-03-04. Red indicates loss or single copy, green indicates gain of copy, and gray indicates loss of heterozygosity on autosomes or two copies of the X chromosome (*i.e.*, female line). (D) Flow cytometry of macrophages differentiated from iPSC with and without SNCA KO (left and right panels, respectively), stained for aSyn (MJFR1 antibody – blue line, isotype control – red line). (E) MS2 extracted ion chromatographs for five peptide precursors of aSyn quantified in SNCA-KO (n = 4) and healthy control (WT; n = 4) iPSC lines. The color coding of the fragments is indicated.

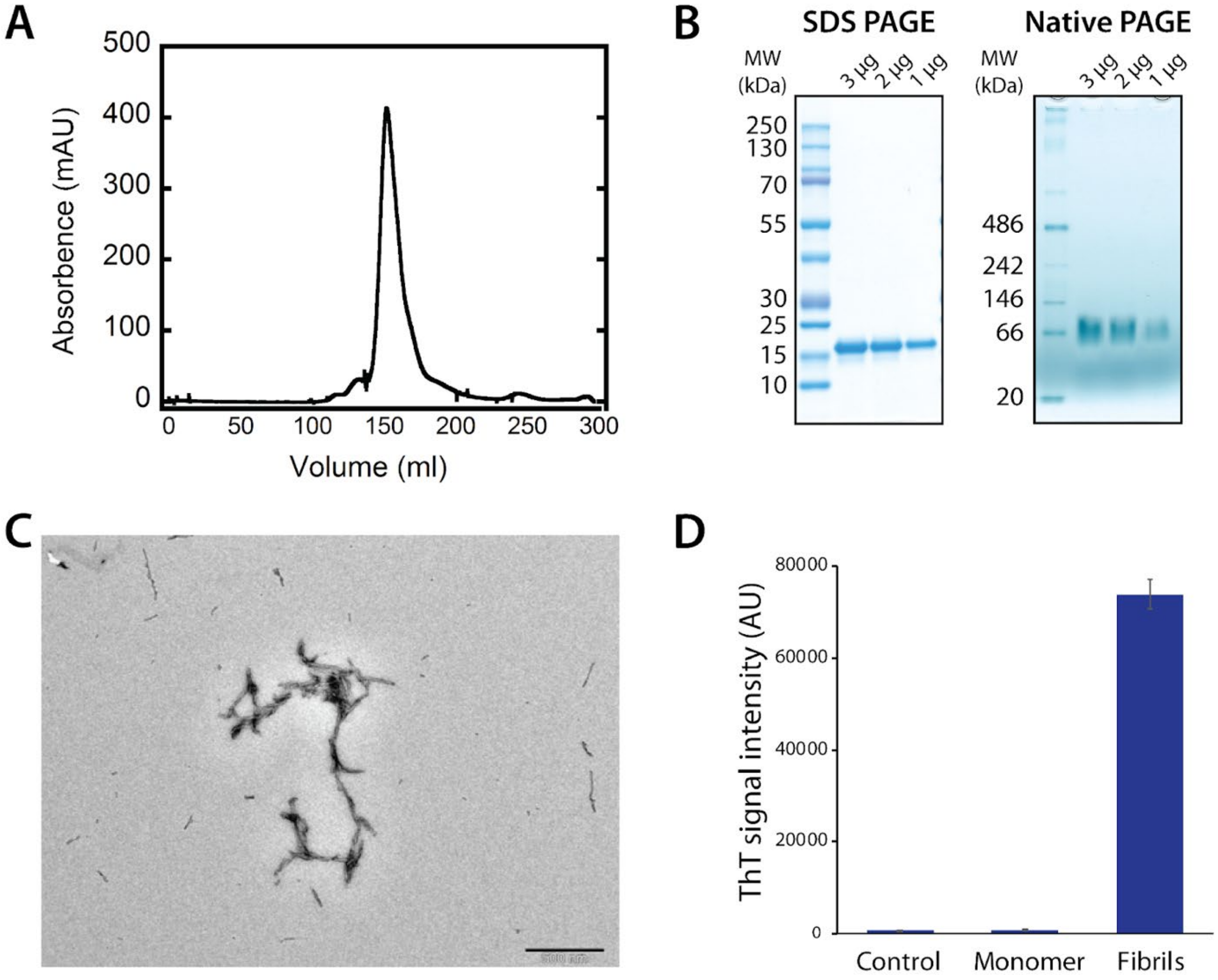

Appendix Figure S4: Quality control experiments for aSyn monomer and amyloid fibrils. (A) SEC analysis of purified aSyn monomer. (B) SDS PAGE and Native PAGE of monomeric aSyn. (C) TEM image of aSyn amyloid fibrils. (D) ThT intensity of PBS control, aSyn monomer, and aSyn amyloid fibrils (n=3 replicates each).

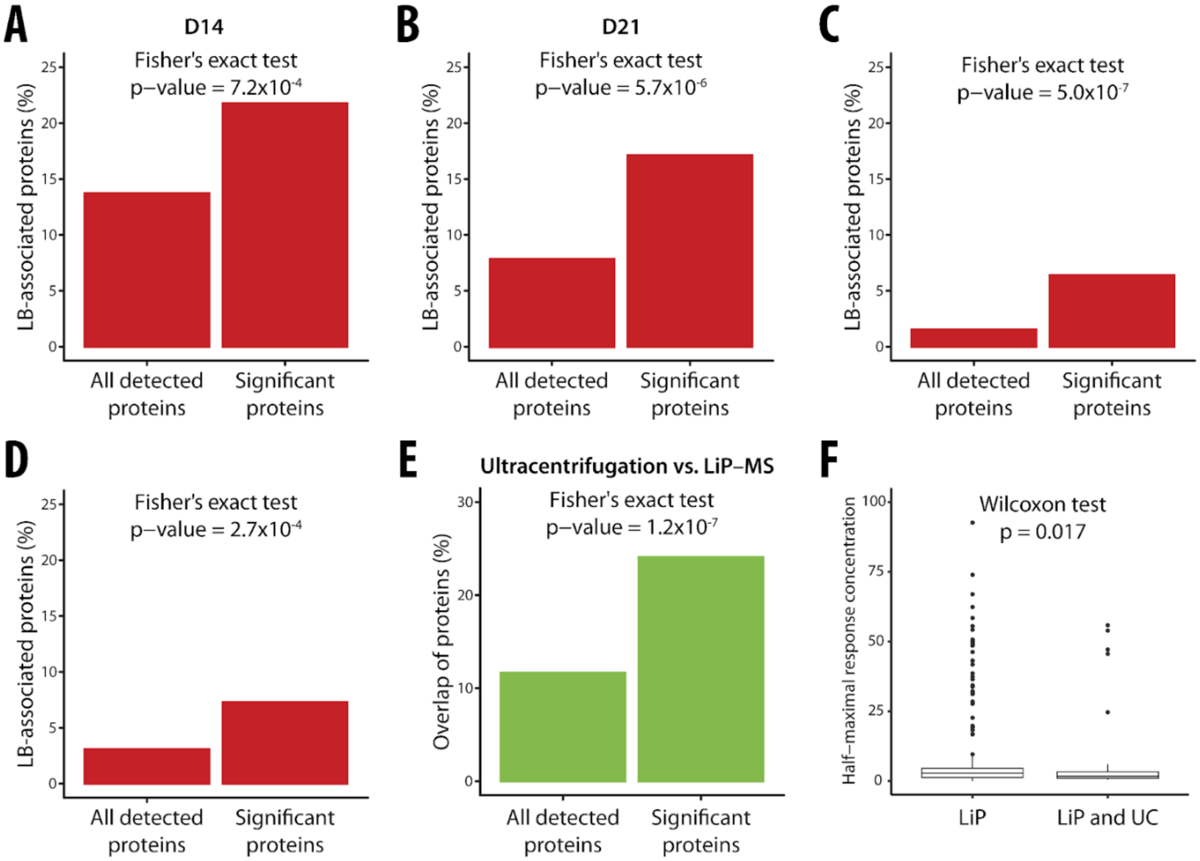

Appendix Figure S5: Identification of Lewy body (LB)-associated and precipitated proteins. (A-D) and fibril-binding proteins by ultracentrifugation (E). (A, B) Enrichment analyses of putative LiP–MS-identified fibril-binding proteins for proteins previously identified as LB components in a neuronal seeding model at two-time points (A, day 14; B, day 21) (Mahul-Mellier *et al*, 2020). The plots show the fraction of previously identified LB components in structurally altered proteins (right bar) versus all detected proteins (left bar) upon spike-in of aSyn fibril into an iPSC-derived cortical neuron lysate. *p*-values assessing enrichment (Fisher's exact test) are shown. (C, D) Enrichment analysis as in A, B for proteins detected in patient-derived LBs (Xia *et al*, 2008) (C) and for proteins identified in patient-derived LBs with neuronal loss (Petyuk *et al*, 2021). (E) Enrichment analysis of putative LiP–MS-identified fibril-binding proteins for ultracentrifugation-identified putative fibril binders. The plots show the fraction of ultracentrifugation-identified fibril binders in structurally altered proteins (right bar) versus all detected proteins (left bar) upon spike-in of aSyn fibril into an iPSC-derived cortical neuron lysate. *p*-values assessing enrichment (Fisher's exact test) are shown. (F) The box plots compare the distribution of half-maximal response concentrations for proteins identified as aSyn fibril candidate interactors by LiP– -MS alone versus proteins identified by both LiP–-MS and ultracentrifugation. The median (line within box), interquartile range (box), potential outliers (individual data points), and *p*-value (Wilcoxon test) are shown.

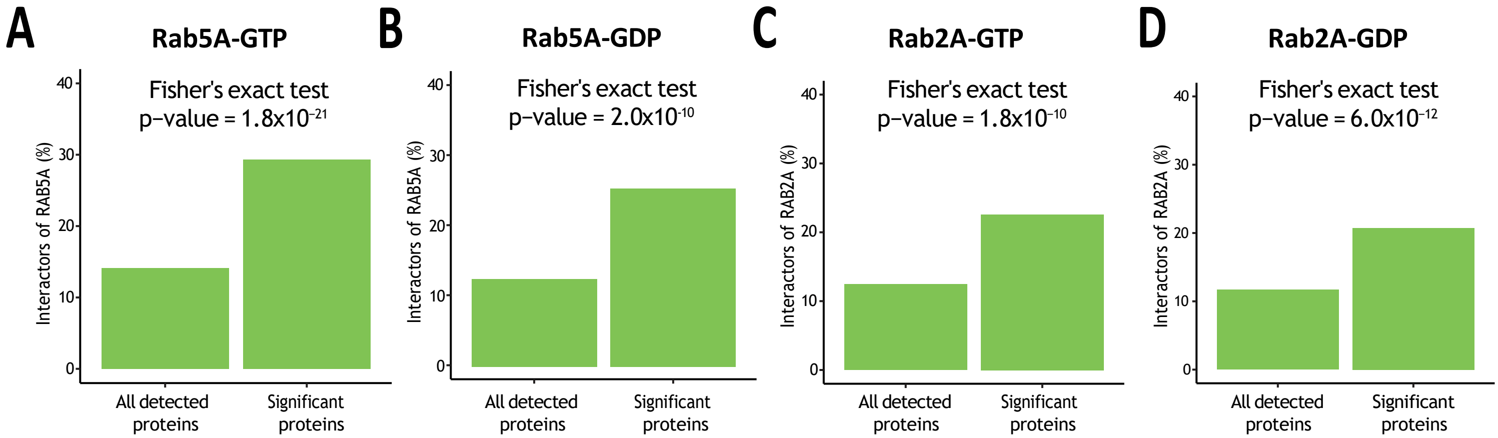
A
Rab5A-GTP
Fisher's exact test
p−value = 1.8x10^-21
Interactors of RAB5A (%)
0
10
20
30
40
All detected proteins
Significant proteins
B
Rab5A-GDP
Fisher's exact test
p−value = 2.0x10^-10
Interactors of RAB5A (%)
0
10
20
30
40
All detected proteins
Significant proteins
C
Rab2A-GTP
Fisher's exact test
p−value = 1.8x10^-10
Interactors of RAB2A (%)
0
10
20
30
40
All detected proteins
Significant proteins
D
Rab2A-GDP
Fisher's exact test
p−value = 6.0x10^-12
Interactors of RAB2A (%)
0
10
20
30
40
All detected proteins
Significant proteins

Appendix Figure S6. Comparison of candidate Rab interactors with results of a previous MitoID screen. The plots show the fraction of MitoID hits in the set of LiP–MS candidate interactors (right) versus all detected proteins (left) upon spike-in of the indicated proteins into a HEK293 cellular extract. The *p*-value assessing enrichment (Fisher's exact test) is shown (n = 4 replicates of each spike-in series with 5 concentrations of the tested protein).

Window	Mass-to-charge ratio (*m/z*)	Charge (*z*)	Isolation window (*m/z*)
1	358	2	16
2	373	2	16
3	388	2	16
4	403	2	16
5	418	2	16
6	433	2	16
7	448	2	16
8	463	2	16
9	478	2	16
10	493	2	16
11	508	2	16
12	523	2	16
13	538	2	16
14	553	2	16
15	568	2	16
16	583	2	16
17	598	2	16
18	613	2	16
19	628	2	16
20	643	2	16
21	659	2	18
22	676	2	18
23	693	2	18
24	710	2	18
25	727	2	18
26	744	2	18
27	761	2	18
28	778	2	18
29	795	2	18
30	813	2	20
31	832	2	20
32	851	2	20
33	870	2	20
34	889	2	20
35	908	2	20
36	929.5	2	25
37	953.5	2	25
38	977.5	2	25
39	1006.5	2	35
40	1048	2	50
41	1111	2	78

Appendix Table S1: Definition of variable windows for DIA–MS measurements.
